# Ecodesign Enhancement of Polymeric Resins: Reinforcing with Synthetic and Natural Fibers Using Theory of Inventive Problem Solving-Algorithm of Inventive Problem Solving for Sustainable Composite Design

**DOI:** 10.3390/polym16243458

**Published:** 2024-12-10

**Authors:** Dan Dobrotă, Cristina Vasilica Icociu, Sergiu Lazăr, Sever-Gabriel Racz, Gina-Maria Moraru

**Affiliations:** 1 Faculty of Engineering, Lucian Blaga University of Sibiu, 550024 Sibiu, Romania; sergiu.lazar@ulbsibiu.ro (S.L.); gabriel.racz@ulbsibiu.ro (S.-G.R.); gina.moraru@ulbsibiu.ro (G.-M.M.); 2 Department of Robots and Production Systems, Faculty of Industrial Engineering and Robotics, National Unversity of Science and Technology Politehnica Bucharest, 060042 Bucharest, Romania; cristina.icociu@upb.ro

**Keywords:** polymeric composites, tensile strength enhancement, ecodesign, natural fiber reinforcement, TRIZ-ARIZ methodology, sustainable materials, mechanical performance optimization

## Abstract

This study examines the enhancement of the mechanical strength of polymer resins through reinforcement with synthetic (glass) and natural (hemp, jute) fibers, using the TRIZ-ARIZ methodology to optimize composite design for improved mechanical properties, sustainability, and economic efficiency. Mechanical testing, thermogravimetric analysis (TGA), and scanning electron microscopy (SEM) were conducted to evaluate the properties of the composite materials. Regarding tensile strength testing, the results showed the following: jute fiber achieved the best results, with a maximum tensile values of 43.75 MPa (partial reinforcement) and 43.53 MPa (complete reinforcement); glass fiber recorded maximum tensile values of 34.55 MPa (partial reinforcement) and 34.52 MPa (complete reinforcement); and hemp fiber yielded maximum tensile values of 24.98 MPa (partial reinforcement) and 24.86 MPa (complete reinforcement). The mechanical performance of partial reinforcements (in the area of maximum stress) was similar to that of complete reinforcements, enabling a reduction in material usage by up to 60%. The thermal analysis (TGA) results demonstrated that glass fiber-reinforced composites exhibit high thermal stability, with mass loss starting at 320 °C and a residual mass of 8.02%; for other composite materials, thermal degradation begins at 305 °C, with a residual mass of 3.69%; in jute fiber-reinforced composites, thermal degradation starts at 300 °C, with a residual mass of 3.71%. SEM analysis generally revealed good fiber–matrix adhesion, while defects such as voids or detached fibers contributed to reduced mechanical strength. These results demonstrate that natural fiber-reinforced composite materials, particularly those reinforced with jute, can be used in sustainable engineering applications. They also show that localized reinforcement provides high performance with minimal resource consumption.

## 1. Introduction

The pervasive use of polymeric materials in modern engineering and manufacturing stems from their versatility, lightweight nature, and cost-effectiveness [1]. Fiber-reinforced composites have emerged as a promising solution, leveraging the synergistic effects of combining polymers with reinforcing fibers [2]. By applying TRIZ-ARIZ principles, we intend to optimize composite structures for improved mechanical performance while considering economic and ecological benefits [1,2,3]. The inherent properties of these resins, such as their low density, corrosion resistance, and ease of processing, make them attractive for various industries, including automotive, aerospace, and construction industries [4]. Mechanically, polymeric resins exhibit properties such as high tensile strength, elasticity, toughness, and hardness, which are essential for structural applications. Despite their advantages, pure polymeric resins often lack the necessary strength and stiffness required for high-load-bearing applications, limiting their use where mechanical demands are substantial [3,5,6].

Tensile strength is a critical parameter that defines a material’s ability to withstand axial stretching forces without failure. In polymer applications, tensile strength determines the material’s capacity to perform under load without experiencing deformation or rupture [7,8]. Enhancing the tensile strength of polymers extends their applicability, allowing for weight reduction, improved performance, and potential cost savings [6,9,10].

These limitations include a relatively low tensile strength and modulus of elasticity compared to metals and ceramics, susceptibility to creep under sustained loads, and degradation under environmental factors such as UV radiation and elevated temperatures [11]. Among the various types of composites, fiber-reinforced composites (FRCs) have garnered significant attention due to their exceptional mechanical properties and versatility in applications [8,12]. The development of FRCs was driven by the need for materials that combine high strength-to-weight ratios, durability, and design flexibility. The ability to engineer composites with specific properties by selecting appropriate fiber and matrix combinations makes FRCs an attractive choice for advanced engineering applications [5,13,14]. The selection of reinforcing fibers in FRCs is a critical decision that impacts the composite’s properties, cost, and environmental footprint. Fibers used in composites can be broadly categorized into synthetic fibers, such as glass, carbon, and aramid fibers, and natural fibers, such as hemp, jute, flax, and sisal [7,15].

Synthetic fibers are manufactured through chemical processes and are known for their high mechanical performance and consistent quality. Glass fibers, for instance, are widely used due to their high tensile strength, good chemical resistance, and relatively low cost. Carbon fibers offer exceptional stiffness and strength-to-weight ratios, making them ideal for high-performance applications like aerospace components [16,17,18].

Natural fibers have emerged as sustainable alternatives to synthetic fibers, driven by environmental concerns and the push for renewable materials. They offer a more environmentally friendly option with the potential to reduce the carbon footprint of composite materials [8,17,18,19,20]. However, natural fibers also present challenges, such as variability in quality, lower thermal stability, moisture absorption, and weaker fiber–matrix adhesion compared to synthetic fibers. Addressing these issues requires careful consideration in composite design and processing techniques [21].

Glass fiber-reinforced composites are among the most extensively studied and utilized FRCs. Glass fibers provide a good balance between mechanical properties and cost, making them suitable for a wide range of applications. Previous studies [5,13,17,19,22,23] have demonstrated that glass fiber reinforcement significantly enhances the tensile strength, stiffness, and impact resistance of polymer matrices.

Research like [16,24,25] has focused on optimizing fiber content, orientation, and surface treatments to improve the interfacial bonding between glass fibers and the polymer matrix. Glass fiber composites are widely used in automotive body panels, wind turbine blades, and marine applications due to their durability and resistance to environmental degradation [26,27].

Hemp fibers have gained attention as a potential reinforcement in polymer composites due to their favorable mechanical properties and ecological benefits. Studies [28,29,30] have shown that hemp fiber composites exhibit improved tensile and flexural properties compared to pure polymer matrices. The specific strength and stiffness of hemp fibers make them competitive with synthetic fibers in certain applications [30].

Research on hemp fiber composites has explored various aspects, including fiber extraction methods, chemical treatments to enhance fiber–matrix adhesion, and the effects of fiber orientation and content on mechanical properties [31]. Challenges such as moisture absorption and variability in fiber quality have been addressed through treatments like alkali treatment, silane coupling agents, and the use of compatibilizers in the matrix [27,28]. Hemp fiber composites are being considered for automotive interior components, construction materials, and consumer goods. Their biodegradability and lower environmental impact align with the growing emphasis on sustainable materials [13,32,33].

Jute fibers are among the most widely available natural fibers, especially in regions like South Asia. Previous studies [17,34,35] have indicated that jute fiber-reinforced composites can significantly improve the mechanical properties of polymers. Jute fibers possess good tensile strength and modulus, making them suitable for reinforcing polymer matrices [19]. Research has explored the use of jute fibers in thermoset and thermoplastic composites. Chemical treatments, such as alkali treatment and acetylation, have been employed to enhance the fiber–matrix interface and reduce moisture absorption. Studies have shown that jute fiber composites exhibit acceptable mechanical properties for applications in packaging, automotive interiors, and low-load structural components [29].

TRIZ (Teoriya Resheniya Izobretatelskikh Zadach) is a theory of inventive problem solving. ARIZ (Algorithm of Inventive Problem Solving) is a structured methodology within TRIZ that provides a systematic approach to solving complex engineering problems. It posits that innovative solutions follow certain principles and that these can be applied to solve problems across different fields. TRIZ provides tools such as the 40 inventive principles, the contradiction matrix, and standard solutions to guide engineers in developing creative and effective solutions [36,37,38]. ARIZ, as an algorithmic component of TRIZ, offers a step-by-step process for defining problems, identifying contradictions, and applying inventive principles to resolve them [39].

In material engineering, the TRIZ-ARIZ methodology can be applied to address challenges such as enhancing mechanical properties, reducing material usage, and improving manufacturing processes [40,41].

In this study, several TRIZ inventive principles are particularly relevant to enhancing the tensile strength of polymer composites:

1 Segmentation (Principle 1): Segmentation involves dividing a system into independent parts, which can improve flexibility, ease of repair, and adaptability.
2 Composite Structures (Principle 40):

This principle suggests combining multiple materials to create a composite that leverages the strengths of each component. In the context of this study, reinforcing the polymer matrix with fibers creates a composite material with improved mechanical properties.

3 Local Quality (Principle 3):

Local quality involves modifying a system non-uniformly to improve performance where needed. Instead of applying a uniform solution throughout the entire system, specific areas are optimized.

4 Continuity of Useful Action (Principle 20):

This principle focuses on ensuring that a useful action is carried out continuously or that all parts of a system are utilized effectively.

By applying these principles, this study aims to develop composite materials that not only exhibit enhanced tensile strength, but also offer benefits in terms of material efficiency, cost reduction, and environmental sustainability. The primary objective of this study is to enhance the tensile strength of polymeric resins through reinforcement using both synthetic (glass) and natural fibers (hemp and jute). By applying the TRIZ-ARIZ principles, this research seeks to optimize the structural integrity of polymer composites, providing sustainable, high-performance alternatives suitable for high-stress applications while addressing ecological concerns.

The manuscript is structured as follows: the introductory Section 1 presents an overview of polymeric materials, composite applications, and TRIZ-ARIZ methodologies. Following this, Section 2 describes the materials and methods employed, including details on specimen preparation and testing protocols. The results are presented in Section 3 of the tensile strength tests, thermogravimetric analysis, and microstructural evaluations. Section 4 provides an analysis of the results, exploring implications for ecological design and proposing refinements based on TRIZ-ARIZ strategies, and in Section 5, the final conclusions regarding the results obtained in the research are presented.

## 2. Materials and Methods

### 2.1. Materials

The materials utilized in this study were carefully selected to achieve the objectives of enhancing tensile strength, exploring localized reinforcement, and assessing ecological and economic benefits. The primary components included a two-component polymeric resin and three types of reinforcement fibers: glass fibers (synthetic) and natural fibers comprising hemp and jute.

#### 2.1.1. Polymeric Resin

The matrix material chosen was Pro-Klar, a two-component polymeric resin supplied by Vosschemie GmbH, Uetersen, Germany. Epoxy Pro Klar is a solvent-free, transparent, and self-leveling two-component epoxy resin produced by Vosschemie GmbH, Germany. Key characteristics: mixing ratio—100 parts resin (component A) to 40 parts hardener (component B) by weight; pot life—approximately 240 min at 23 °C, appearance—clear and transparent; consumption—about 1.1 kg per liter. This resin was selected due to its excellent mechanical properties, including a Shore D hardness of 75, which indicates a suitable balance between rigidity and toughness for structural applications. The resin’s curing characteristics, such as a manageable pot life and curing time, facilitated efficient specimen preparation without compromising fiber integrity. Additionally, Pro-Klar cures to a clear finish, allowing for visual inspection of fiber distribution and potential defects within the composite specimens. Its compatibility with various fibers ensures effective adhesion, which is essential for load transfer in fiber-reinforced composites.

#### 2.1.2. Reinforcement Fibers

The glass fibers used in this study are commercial-grade E-glass chopped strands (6–8 mm length), procured from SC Best TOOLS SRL, a local supplier, chosen for their high tensile strength, chemical resistance, and cost-effectiveness. Natural fibers, specifically hemp and jute, were sourced directly from local producers in regional markets, emphasizing sustainability and eco-friendliness. Hemp fibers, trimmed to 5–10 mm, were derived from industrial hemp plants, while jute fibers underwent similar manual preparation. All fibers were dried at 80 °C for 24 h to minimize moisture content and ensure optimal adhesion with the Pro-Klar resin. To maintain uniformity, the fiber volume fraction was 30% ± 5%, with an amorphous distribution within the matrix to achieve isotropic mechanical properties. These specifications ensure that future researchers can replicate the methodology by sourcing comparable materials and adhering to the detailed fiber preparation and composite fabrication protocols outlined.

The selection rationale focused on comparing synthetic and natural fibers to evaluate the trade-offs between mechanical performance and environmental impact. Natural fibers offer advantages such as lower energy consumption during production, biodegradability, and reduced carbon footprint. Assessing different fiber types, we aimed to identify suitable materials for applications where ecological and economic factors are significant.

### 2.2. Specimen Preparation

**Figure 1:**
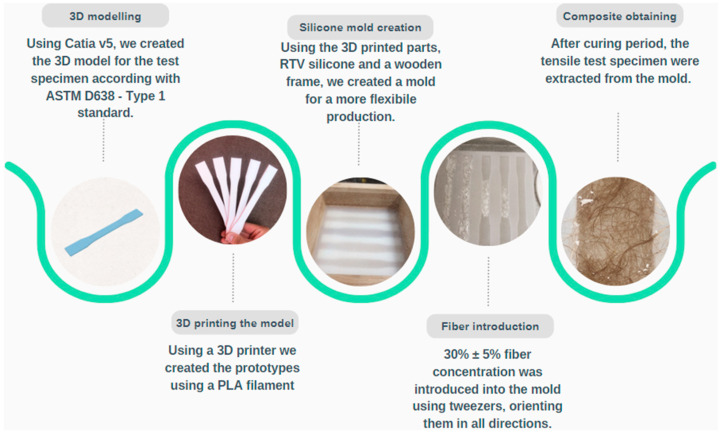
Tensile test specimen creation steps.

Consistent and controlled specimen preparation was crucial for obtaining reliable and comparable results. All specimens were prepared following the ASTM D638 Type I standard for tensile testing [42]. The generic roadmap for creation is available in Figure 1 below: 

#### 2.2.1. Pure Polymeric Specimens

For the fabrication of pure polymeric specimens, silicone molds corresponding to the ASTM D638 Type I dimensions were created to ensure accurate geometry and surface finish. The dimensions of the samples were as follows: overall length—165 mm; gage length—50 mm; width at narrow section—13 mm; length of reduced section—57 mm; width of grip section—19 mm; length of grip Section—50 mm; thickness—5 mm. The two components of the Pro-Klar resin were mixed thoroughly according to the manufacturer’s recommended weight ratio to achieve homogeneity. The mixed resin was degassed under vacuum to eliminate air bubbles that could act as stress concentrators. The resin was then carefully poured into the silicone molds to prevent air entrapment.

Curing was performed at room temperature for 72 h, followed by post-curing at 60 °C for 2 h to ensure full polymerization. After curing, the specimens were gently demolded. To maintain replicability, all specimens were prepared in a controlled laboratory environment with constant temperature and humidity. Visual inspections were conducted to ensure specimens were free from defects such as voids, cracks, or irregularities.

#### 2.2.2. Composite Specimens

Composite specimens were prepared with a targeted fiber volume fraction of 30% ± 5%, a content selected based on the literature indicating optimal reinforcement without excessive viscosity or processing difficulties. The fibers were manually oriented in three spatial directions to create an amorphous structure, aiming for isotropic mechanical properties.

The fibers were dried in an oven at 80 °C for 24 h to reduce moisture content, which could affect adhesion and composite properties. The required amount of fibers for each specimen was weighed to achieve the desired fiber content. The Pro-Klar resin components were mixed as per the pure specimen preparation.

The fibers were gradually and evenly distributed into the mold with tweezers, taking care to prevent fiber agglomeration or voids. The fiber–resin mixture was then poured into the silicone molds, and gentle vibration was applied to assist in fiber settling and the removal of trapped air. Curing conditions identical to those for the pure polymeric specimens were maintained. After curing, the specimens were carefully demolded to prevent damage.

To ensure consistency, the same technician performed all specimen preparations, and detailed records of material quantities, mixing times, and environmental conditions were maintained.

### 2.3. Application of TRIZ-ARIZ Principles

The TRIZ-ARIZ principles were applied in designing and fabricating composite specimens to explore innovative reinforcement strategies, including composite structures, local quality, segmentation, and preliminary anti-action.

#### 2.3.1. Composite Structures

**Figure 2:**
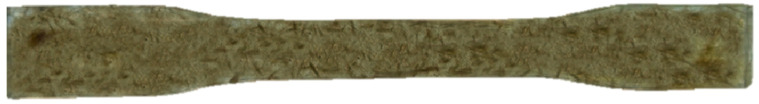
Tensile test specimen fully reinforced with hemp fiber.

In applying the principle of composite structures, the initial reinforcement involved incorporating fibers throughout the entire specimen to evaluate the maximum potential enhancement in tensile strength provided by full reinforcement with each fiber type, as shown in Figure 2 below.

Composite specimens were prepared as previously described, with fibers distributed uniformly. Five specimens were fabricated for each fiber type—glass, hemp, and jute—to ensure statistical validity. These specimens were subjected to tensile testing according to ASTM D638, and data on breaking force, tensile strength, and elongation at break were recorded. The results were compared to assess the relative performance of each fiber type in full reinforcement.

#### 2.3.2. Local Quality—Narrow Side Reinforcement

**Figure 3:**
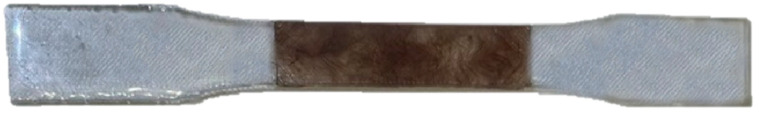
Tensile test specimen partial reinforced with jute fiber.

Applying the principle of local quality involved reinforcing only the narrow section of the ASTM D638 Type I specimen, corresponding to the gauge length where maximum stress occurs during tensile loading. The representative specimen for this applied principle is shown in Figure 3.

Fibers were incorporated only in this region, while the wider grip sections consisted of pure polymeric resin. The same fiber content of 30% ± 5% was maintained within the reinforced region. Five specimens were prepared for each fiber type.

The reduction in fiber usage and total specimen mass was calculated based on the volume difference between full reinforcement and localized reinforcement, anticipating material savings and weight reduction. During fabrication, fibers were strategically placed within the narrow region, and transitions between reinforced and unreinforced regions were smoothed to avoid stress concentrations. Identical tensile testing procedures were applied to these specimens.

#### 2.3.3. Segmentation

**Figure 4:**
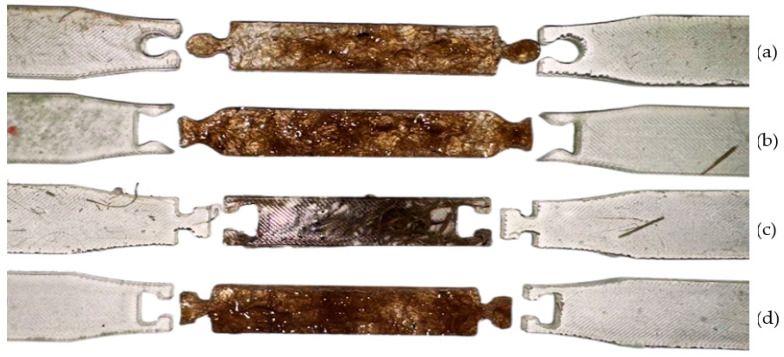
Segmentation of the tensile test specimen. (**a**) Puzzle, (**b**) dovetail, (**c**) T-channel1, (**d**) T-channel 2.

Incorporating the principle of segmentation, the specimens were divided into three segments: two grip sections and a central gauge section, as detailed in Figure 4 below. Mechanical assembly methods were employed, including puzzle joints with interlocking shapes, dovetail joints allowing for sliding assembly, and T-channels with slots and protrusions forming a ‘T’ shape for alignment. The rationale for this approach was to avoid the use of adhesives, facilitating recycling and reducing chemical usage, as well as enhancing repairability by allowing damaged sections to be replaced without discarding the entire specimen.

Each segment was cast separately using silicone molds designed with the specific joint geometry. The central gauge section was reinforced with fibers as previously described, while the grip sections were composed of pure polymeric resin. After curing, segments were assembled manually, ensuring a snug fit to maintain structural integrity during testing. The assembled specimens were inspected for gaps or misalignments before undergoing tensile testing under the same conditions as the other specimens.

**Figure 5:**
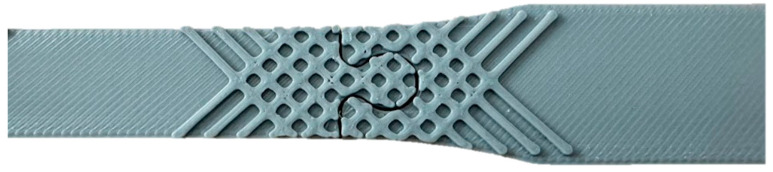
Another dimension—reinforcement using ribs.

To enhance the stiffness of the component while maintaining a uniform clearance of 0.5 mm around each part, we decided to modify the standard design by applying the TRIZ inventive principle known as “Another Dimension.” This principle involves introducing an additional feature or element to the design to improve its performance characteristics. The addition dimension added means also that we do not involve anything related to the fiber or structure, but the modification of the part thickness in order to improve the general behavior. The modified design incorporates this generic concept, as depicted in Figure 5.

#### 2.3.4. Preliminary Anti-Action

**Figure 6:**
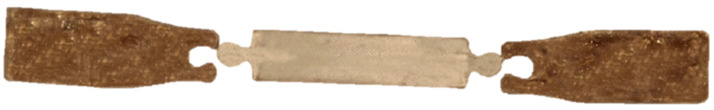
Anti-action of the tensile test specimen.

To assess the necessity of reinforcement in the stressed region, the principle of preliminary anti-action was applied by reinforcing only the grip areas, as is illustrated in Figure 6. Thus, fibers were incorporated solely in the grip sections, leaving the narrow-gauge section as pure polymeric resin. This configuration tested the hypothesis that reinforcement in non-stressed areas would not contribute significantly to tensile strength. It was expected that a significant reduction in tensile strength would occur due to the absence of reinforcement in the maximum stress region. The outcomes of these tests would validate the importance of targeted reinforcement.

### 2.4. Testing Methods

Comprehensive testing methods were employed to evaluate the mechanical performance, thermal stability, microstructural characteristics, and moisture content of the fibers and composites.

#### 2.4.1. Tensile Testing

**Figure 7:**
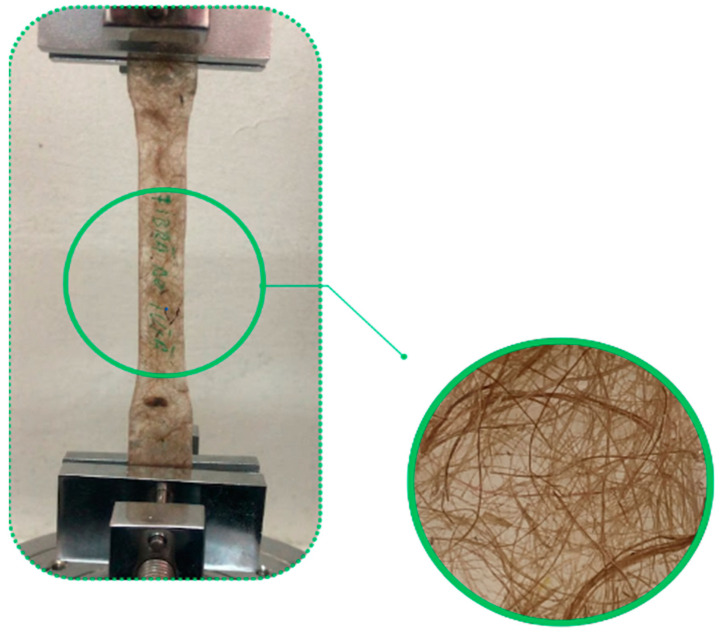
Sauter testing machine with bio composite test specimen.

Tensile testing was conducted using an SAUTER TVM20KN120N Motorized vertical test stand manufacturing SAUTER Group, Basel, Switzerland, which is visible in Figure 7.

The crosshead speed was set at 14.3 mm/min in accordance with ASTM D638 specifications. Load and extension data were recorded continuously during the tests.

Five specimens were tested for each configuration and fiber type to obtain statistically significant results. Prior to testing, specimens were conditioned at 23 °C and 40% relative humidity for 48 h. Dimensions of each specimen, including thickness and width, were measured using a digital caliper manufacturing Mitutoyo, Kawasaki, Japan.

The specimens were clamped securely in the grips of the testing machine to prevent slippage and aligned vertically to avoid bending stresses. Tests were initiated, and data were collected until specimen failure. Tensile strength, modulus, and elongation at break were calculated from the recorded data.

#### 2.4.2. Thermogravimetric Analysis (TGA)

Thermogravimetric analysis was performed using a BAXIT BXT-TGA-1600 thermogravimetric analyzer (Glomro Industrial Co., Ltd., Shanghai, China) to assess the thermal stability and decomposition behavior of the composites. Small specimens weighing approximately 43–64 mg were cut from the composite samples. Tests were conducted under a nitrogen atmosphere to prevent oxidation, with the parameters from Table 1.

Weight loss curves were recorded to identify decomposition temperatures and to compare the thermal stability of the different composites based on the onset of degradation and residual mass.

#### 2.4.3. Scanning Electron Microscopy (SEM)

Scanning electron microscopy was utilized to evaluate the fiber–matrix adhesion and microstructural characteristics of the composites. Samples were taken from the fractured ends of tensile specimens and sputter-coated with a thin layer of gold to prevent charging during imaging. A Phenom Pure G6 Desktop SEM microscope scanning electron microscope (Thermo Fisher Scientific, Waltham, MA, USA) was employed for the analysis. The SEM examination focused on assessing the quality of bonding between fibers and the polymer matrix, identifying defects such as voids, fiber pull-outs, or debonding at the interface, and examining fiber distribution and orientation within the composite.

## 3. Results

### 3.1. Tensile Test Analysis

The tensile testing results of polymer composites reinforced with glass, hemp, and jute fibers reveal several critical findings about the mechanical performance of these materials. Analyzing both the maximum force and average tensile strengths achieved in tests with various fiber configurations offers insight into how targeted reinforcement strategies can significantly enhance the strength and sustainability of composites. These findings, particularly the comparable results between narrow-side reinforcement and full-length reinforcement, have substantial implications for ecodesign, economic efficiency, and potential future applications.

#### 3.1.1. Overview of Tensile Testing Results and Fiber Comparison

The baseline performance of the pure polymer matrix, tested without any fiber reinforcement, highlights the inherent limitations of polymers when subjected to tensile stress, Table 2. With a maximum tensile of 19.2 MPa and an average tensile of 18.78 MPa across five tests, the unreinforced matrix exhibits relatively low tensile strength and limited capacity to withstand high-stress applications. This serves as a control, emphasizing the value added by fiber reinforcements in enhancing the mechanical strength of polymer composites.

Adding fiber reinforcements to the polymer matrix greatly improves tensile strength across all configurations. Glass, hemp, and jute fibers each contribute uniquely to the composites, reflecting their individual mechanical properties and environmental benefits. Glass fiber composites achieve a maximum tensile of 34.55 MPa with narrow reinforcement and 34.52 MPa with full-length reinforcement, showing a substantial increase in tensile strength compared to the pure matrix. Similarly, hemp fiber composites reach maximum tensile values of 24.98 MPa and 24.86 MPa for narrow and full-length reinforcement, respectively, while jute fiber composites perform exceptionally well, achieving maximum tensile values of 43.45 MPa and 43.54 MPa in the narrow and full-length reinforcement configurations.

**Figure 8:**
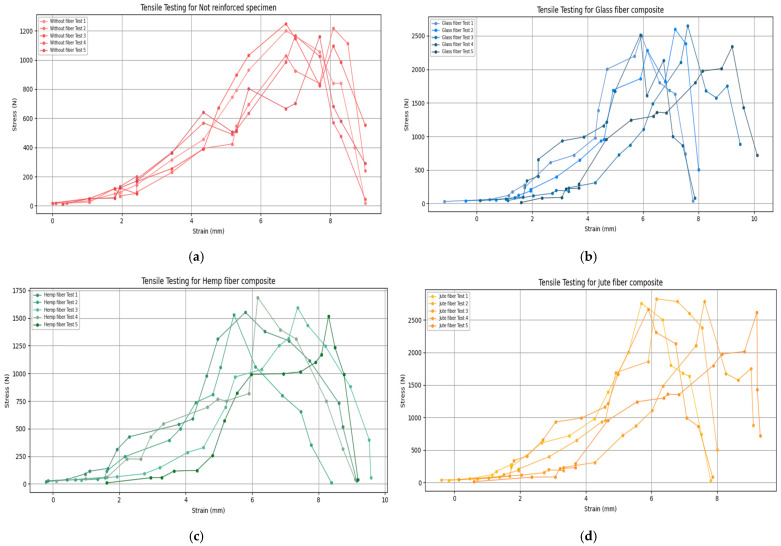
Tensile testing curves for (**a**) non-reinforced material, (**b**) glass fiber-reinforced material, (**c**) hemp fiber-reinforced material, and (**d**) jute fiber-reinforced material.

The graph in Figure 8 depicting the stress–strain behavior of different fiber-reinforced polymer composites highlights an essential relationship between displacement (strain) and the force at which each material reaches its breaking point. Examining the displacement at failure for each fiber type provides insight into the flexibility and ductility of these materials, which is crucial for applications requiring both strength and moderate flexibility.

The displacement-to-breaking force relationship shown in the graph reveals the dual role of fiber reinforcement in enhancing both the tensile strength and flexibility of the polymer matrix. The glass and jute fiber composites achieve higher tensile strengths with moderate elongation before failure, which means they can absorb energy and deform to a certain extent, making them suitable for structural applications that require resilience against dynamic loads. Hemp, though less strong, still provides a moderate level of strength and flexibility, positioning it as a suitable choice for applications where sustainability is prioritized over maximum load-bearing capacity.

In addition to the maximum force and average tensile strength, an analysis of Young’s modulus and strain-to-failure provides further insights into the mechanical performance and flexibility of each composite material. This analysis is depicted in Table 3. The Young’s modulus values, which represent the material’s stiffness, and the strain-to-failure percentages, which indicate ductility, reveal how each fiber type contributes to the overall balance between strength and flexibility in the composites.

The pure polymer matrix exhibits a relatively low Young’s modulus of 2.1 GPa, along with a strain-to-failure of 1.5%. This confirms that, without reinforcement, the matrix is both less stiff and less capable of elongating before failure. In comparison, glass fiber reinforcement significantly enhances the composite’s stiffness, with Young’s modulus values of 5.8 GPa for narrow reinforcement and 5.7 GPa for whole-part reinforcement. This increase in stiffness is paired with a strain-to-failure of around 2.3% to 2.4%, reflecting a moderate flexibility that makes glass-reinforced composites ideal for load-bearing applications requiring resilience.

Hemp fibers contribute moderately to the stiffness of the composite, with Young’s modulus values of 3.9 GPa for narrow-area reinforcement and 3.8 GPa for whole-part reinforcement. The strain-to-failure for hemp-reinforced composites is between 1.9% and 2.0%, indicating a good balance between flexibility and strength that makes hemp suitable for eco-friendly applications where medium tensile strength and ductility are required.

These values show that each fiber type brings unique mechanical advantages, with glass providing high stiffness, jute offering excellent tensile strength and energy absorption, and hemp achieving a balanced performance with sustainable benefits.

**Figure 9:**
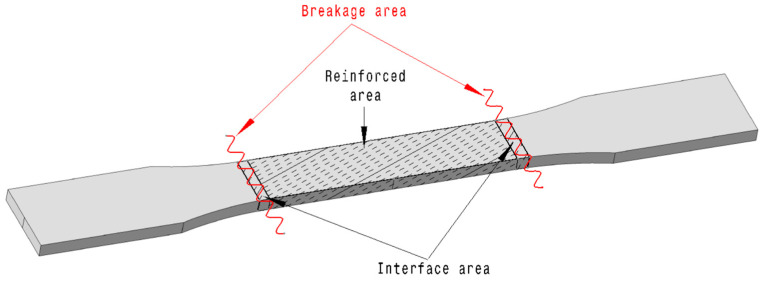
Breaking line for partially reinforced specimen.

Additionally, the results (14 out of 15) show that breakage in the partial fiber-reinforced composites typically occurred at the interface where the fiber-reinforced section meets the adjacent non-reinforced matrix, as depicted in Figure 9. This observation indicates a concentration of stress at the boundary, likely due to the sudden change in material properties such as stiffness and strength between the reinforced and non-reinforced sections. The abrupt transition between these two regions creates a point of vulnerability, where the composite is more likely to experience failure under load. This stress concentration is especially pronounced in areas where the fiber reinforcement tapers off, suggesting that the structural integrity of the composite is influenced not only by the presence of fibers but also by how smoothly the load is transferred across different sections of the material.

The reinforcement applied in the narrow area effectively strengthens that high-stress zone, confirming the benefit of targeted reinforcement in composite design. However, this also shifts the failure point away from the reinforced area to the regions where reinforcement is absent or decreases, emphasizing the need for careful design at these transition points. This finding has significant implications for composite manufacturing, as it highlights the importance of optimizing the interface between reinforced and non-reinforced areas. By carefully managing these transition zones, it is possible to maximize the structural benefits of reinforcement while minimizing the risk of premature failure due to stress concentration.

##### Modular Assembly Design

The results presented in Table 4 provide additional insights into the tensile performance of polymer composites reinforced with glass, hemp, and jute fibers, but configured in modular assembly designs: puzzle, dovetail, and T-channel. These configurations introduce mechanical interlocking mechanisms which add complexity to the composite’s load-bearing behavior compared to the simpler, continuous reinforcement structures discussed previously. The maximum and average forces achieved in each configuration are available in Table 4, and they highlight how fiber type and modular design affect distribution and resistance to tensile stress.

The puzzle configuration, particularly with glass and jute fibers, achieves the highest tensile strength among all modular designs, reaching maximum tensile values of 22.97 MPa for glass and 22.8 MPa for jute. This performance is noteworthy, though still lower than the continuous reinforcement configurations, where glass and jute achieved tensile values of approximately 34.55 MPa and 43.45 MPa, respectively. The puzzle design demonstrates the ability of interlocking features to engage fiber reinforcements effectively, though with some trade-off in maximum tensile strength, likely due to interruptions in fiber continuity.

In contrast, the dovetail and T-channel configurations display lower maximum tensile values, especially in the hemp-reinforced composites, where values are reduced to 17.11 MPa in the dovetail configuration and around 16.11–16.6 MPa in T-channel assemblies. These results suggest that the interruption of fiber continuity in modular designs, while structurally beneficial for assembly and reusability, impacts overall strength due to stress concentrations at joint interfaces.

Fracture analysis reveals that the puzzle and dovetail configurations offer more effective load distribution within the composite, with fractures consistently occurring in the narrow, fiber-reinforced regions. In these configurations, the fibers bear the tensile load directly, allowing the composite to utilize its mechanical properties more fully. This behavior contrasts with the continuous reinforcement structures where the primary breakage occurs at the transition between reinforced and non-reinforced areas due to abrupt property changes. Here, in the puzzle and dovetail configurations, the breakage within the composite-reinforced area indicates that load transfer is optimized within the modular joint, with fibers directly engaged in resisting applied forces up to the failure point. This behavior underscores the value of strategically designed interlocking joints in maximizing load-bearing potential even in segmented composites.

**Figure 10:**
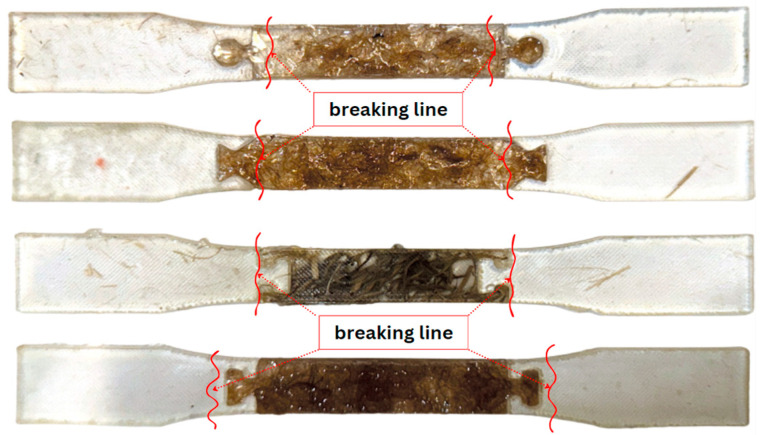
Braking position for three-part test specimen.

In addition, the fracture analysis provides valuable insights into how different modular designs distribute and resist tensile stress, with Figure 10 illustrating distinct fracture lines for each configuration.

The puzzle and dovetail configurations demonstrate a unique effectiveness in engaging the composite material under load, which is confirmed by the consistent fracture patterns observed within the composite-reinforced areas. This contrasts with the T-channel design, where breakage tended to occur at the joint interfaces, suggesting that fiber engagement was less optimized.

**Figure 11:**
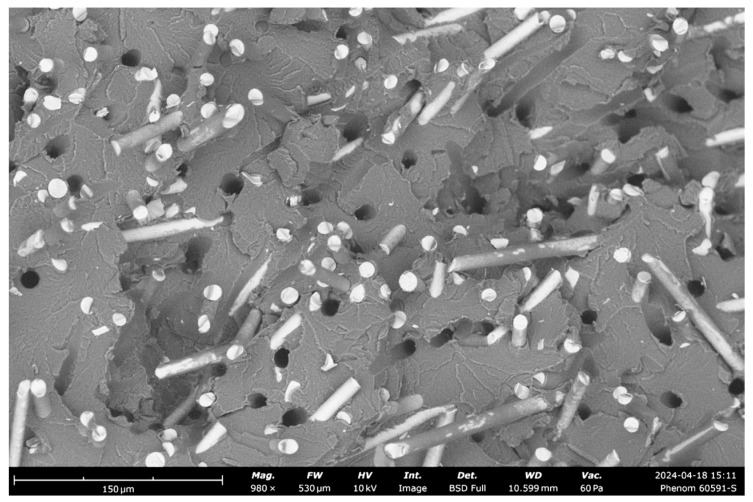
Microstructural analysis of 30% glass fiber-reinforced polymer composites through SEM.

In both the puzzle and dovetail configurations, fiber reinforcements are strategically positioned to directly bear tensile loads, causing fractures to localize in narrow, stress-concentrated regions within the reinforced composite. This fracture pattern, evident in Figure 11, reveals that these configurations maximize the composite’s mechanical properties by allowing the fibers to engage fully in resisting applied forces until failure. The concentrated breakage within the composite area, rather than at joint interfaces, suggests a more efficient load transfer, effectively allowing the material to withstand higher tensile forces before reaching its failure threshold.

This finding highlights the critical role of modular design and fiber placement in enhancing composite performance. Puzzle and dovetail designs not only utilize the reinforcement for stress distribution but also ensure that the composite material’s tensile strength is optimized, making them ideal for applications where load-bearing capacity and material resilience are prioritized. Figure 10 visually confirms this behavior, emphasizing that strategic fiber positioning within modular assemblies significantly influences the overall durability and performance of the composite under tensile stress.

In summary, while modular configurations such as puzzle and dovetail achieve lower maximum forces than continuous reinforcement, they demonstrate an efficient distribution of stress across the jointed areas, particularly when paired with high-strength fibers like glass and jute. This modular approach enhances structural integrity through interlocking features that engage fibers under load, although at the cost of some tensile strength. The T-channel configurations, while structurally sound, appear less efficient in distributing stress, likely due to the less direct engagement of the fibers under tensile load, as indicated by lower maximum forces and earlier onset of failure. These findings suggest that modular composite designs, especially with puzzle and dovetail configurations, could be advantageous in applications requiring disassembly or repair while maintaining a reasonable level of tensile performance, particularly if paired with high-performance fibers like glass or jute.

When failure occurred outside the reinforced region, we observed that the mechanical performance was closely aligned with that of the pristine material. However, this research focused on specimens that failed in the reinforced zone, as they are the most representative of the strengthening effect of the material.

*Modular assembly design—adding another dimension*

For the modular parts shown in Figure 10, tensile tests were conducted under identical parameters to evaluate the effect of additional rib reinforcements. These parts were reinforced with 1 mm square ribs strategically placed in areas identified as particularly sensitive to stress. The introduction of these ribs resulted in a notable improvement, visible in Table 5, increasing tensile strength by approximately 20%, which reflects a significant enhancement in stiffness and overall structural integrity.

For example, ribbed glass fibers in the puzzle configuration achieved a maximum tensile value of 26.87 MPa, significantly higher than the 22.97 MPa recorded in the non-reinforced version. Similarly, the ribbed jute puzzle configuration achieved a maximum tensile value of 26.90 MPa, indicating a marked improvement over the unreinforced maximum of 22.8 MPa. The observed increase in tensile strength highlights the enhanced stiffness and overall structural integrity of ribbed modular composites. The incorporation of ribs plays a critical role in stress management, effectively dispersing loads across the composite structure. This mechanism delays the onset of failure by reducing localized stress concentrations, ensuring more uniform load distribution throughout the material. In particular, the puzzle and dovetail configurations demonstrate superior resistance to tensile stresses due to their mechanical interlocking features. These configurations capitalize on the inherent strength of modular designs, compensating for the discontinuity in fiber alignment that typically weakens composite structures. The ribs in these assemblies not only strengthen the material, but also ensure a robust connection between modules, making them highly efficient in maintaining structural coherence under tensile loads. This improvement in performance underlines the versatility of ribbed modular composites, particularly in applications demanding high durability, strength, and reusability. The mechanical interlocking provided by ribbed designs enhances load-bearing capacity while maintaining the flexibility needed for disassembly and reconfiguration. Such attributes make these composites particularly well suited for industries where modularity, ease of repair, and adaptability are crucial, such as the aerospace, automotive, and construction industries. The ability to balance structural integrity with assembly flexibility offers a compelling advantage, ensuring both reliability and versatility in demanding use cases.

The puzzle and dovetail configurations are specifically designed to manage stress effectively in jointed areas. Their interlocking features allow for even load transfer—tensions are distributed more uniformly, reducing the likelihood of stress concentration zones that can lead to premature failure; and enhanced structural stability—the interlocking mechanism ensures that the modular components remain securely connected under load, even when fiber continuity is interrupted. Glass fibers significantly improve the performance of modular joints by absorbing and redistributing applied stresses. Jute fibers possess considerable strength-to-weight ratios, making them a cost-effective and sustainable reinforcement option. When combined with modular configurations, they enhance the overall mechanical behavior without compromising sustainability. The modular design inherently involves interruptions in fiber continuity, which leads to a reduction in tensile strength compared to continuous reinforcements. However, the mechanical interlocking compensates for this by engaging the fibers effectively under load, preserving structural integrity in demanding applications.

Unlike puzzle and dovetail designs, T-channel configurations exhibit less efficient stress distribution. This inefficiency is likely due to reduced fiber engagement—the structural geometry of T-channel designs limits the direct involvement of fibers under tensile loads, resulting in lower stress transfer efficiency; and localized stress concentrations—these regions are more prone to failure, as indicated by the earlier onset of failure and lower maximum forces recorded during testing. Although T-channel configurations maintain basic structural soundness, they are less effective at handling high tensile forces, making them less suitable for applications requiring high performance under sustained or extreme loads. The modular nature of puzzle and dovetail configurations provides a significant advantage in applications that demand frequent maintenance, repair, or reassembly. Their design allows for ease of replacement—individual modules can be replaced or repaired without compromising the entire structure; reusability—modular components can be disassembled and reused, aligning with sustainable engineering practices. While modular configurations sacrifice some tensile performance compared to continuous reinforcements, they achieve an optimal balance between structural integrity and flexibility. This makes them ideal for applications where adaptability is crucial. The use of high-performance fibers like glass and jute in modular composites helps offset the reduction in tensile strength, ensuring these configurations remain viable. The findings indicate that modular composite designs, especially those employing puzzle and dovetail configurations, offer a practical and innovative solution for applications requiring durability, modularity, and repairability. These configurations excel in distributing stresses across jointed areas and maintaining structural integrity under load, particularly when reinforced with high-strength fibers like glass and jute. While T-channel designs are structurally sound, their lower efficiency in stress distribution and earlier failure onset highlight the superiority of puzzle and dovetail designs in applications demanding both strength and modular flexibility. These attributes position modular composites as a promising choice in industries such as aerospace, automotives, and construction, where the balance between performance and adaptability is critical.

##### Modular Assembly Design—Adding Anti-Action

The tensile performance of composite specimens reinforced using an anti-action approach is evaluated. Here, fibers are placed in the wider, less stressed sections, avoiding the narrow regions that experience higher stress concentrations during tensile loading. This placement impacts load-bearing capacity, as fibers are absent in critical areas, reducing overall tensile strength, as can be proven by Table 6.

Comparative data show that the anti-action design consistently achieves lower maximum and average tensile values than segmented designs, especially those with ribs. For instance, in the puzzle configuration with glass fibers, the anti-action design reaches a maximum tensile value of 15.25 MPa, compared to 22.97 MPa in the segmented design and 26.88 MPa in the rib-reinforced version. Similar reductions are observed in hemp and jute configurations, highlighting the importance of fiber placement in high-stress zones.

The anti-action tests, where fibers were limited to the wider grip sections, confirmed the hypothesis that reinforcement in non-stressed areas does not significantly enhance tensile strength. The results showed a marked reduction in tensile capacity compared to configurations with fibers in high-stress regions, validating the need for targeted reinforcement in the narrow, high-stress areas to maximize load-bearing performance. These findings highlight that strategic fiber placement in modular composites is essential for optimizing tensile strength and structural integrity.

#### 3.1.2. Key Discovery: Comparable Performance of Narrow and Whole-Part Reinforcement

One of the most significant findings in this study is the minimal difference in tensile strength between composites with narrow-side reinforcement and those with full-length reinforcement. Across all fiber types, the strength values are remarkably similar between these two configurations. For example, glass fiber composites achieve almost identical maximum forces in both configurations (34.55 MPa for narrow reinforcement and 34.52 MPa for full-length reinforcement). The hemp and jute composites follow this trend, with tensile strengths differing by only a few Newtons between the narrow and full-length configurations. This similarity suggests that strategically placing fibers in the narrow (high-stress) regions of the composite is nearly as effective as reinforcing the entire length, a discovery with profound implications.

From an economic perspective, the ability to achieve comparable mechanical performance with narrow reinforcement translates directly into cost savings. Full-length reinforcement requires more fiber material, which increases the overall material cost of the composite. By concentrating reinforcement only in critical stress regions, it is possible to reduce the amount of fiber needed without sacrificing structural integrity. For industries where production costs are a key concern—such as automotive, aerospace, and construction industries—this targeted reinforcement approach offers a means of reducing raw material expenses, which can significantly lower production costs on a large scale.

Ecologically, the narrow-side reinforcement strategy aligns well with principles of sustainable design by minimizing resource consumption. The use of natural fibers like hemp and jute in composite applications already offers ecological benefits, as these fibers are renewable, biodegradable, and less resource-intensive than synthetic fibers like carbon or glass. Narrow reinforcement further enhances the sustainability profile by reducing the amount of fiber used in each composite. For example, using jute or hemp only in the narrow section of a polymer composite can reduce fiber consumption by up to 60%, leading to a lower carbon footprint, less energy expenditure during production, and less waste generated in the manufacturing process. This discovery supports an ecodesign approach, where material efficiency is prioritized alongside mechanical performance.

#### 3.1.3. Implications for Ecodesign and Sustainable Engineering

Ecodesign principles focus on minimizing environmental impact throughout a product’s lifecycle, from raw material sourcing to disposal or recycling. The findings from this study underscore the potential of polymer composites with narrow-side reinforcement to advance sustainable engineering practices. By applying TRIZ’s “Local Quality” principle, which emphasizes the targeted use of materials where they are most needed, this approach maximizes resource efficiency. The narrow reinforcement strategy enables manufacturers to produce strong, durable composites with fewer materials, aligning with the core tenets of ecodesign.

This reinforcement approach also complements the broader goals of sustainable development, as outlined in initiatives like the United Nations’ Sustainable Development Goals (SDGs). SDG 12, which calls for responsible consumption and production, is directly addressed by reducing material inputs without compromising product performance. Furthermore, by reducing the dependency on synthetic fibers, which are derived from non-renewable resources, this approach supports SDG 13, focused on climate action, by lowering the environmental impact associated with fiber production and composite manufacturing.

#### 3.1.4. Potential Applications and Future Directions

The findings from this study have several implications for future applications of polymer composites. Industries that rely on high-strength, lightweight materials, such as automotive, aerospace, marine, and construction, stand to benefit significantly from the narrow-side reinforcement strategy. In automotive applications, for example, fiber-reinforced composites are used to manufacture lightweight body panels, interior components, and structural elements that contribute to fuel efficiency and overall vehicle performance. The cost and material savings from narrow reinforcement could make these composites more accessible for mass-market vehicles, supporting a broader adoption of lightweight materials to reduce vehicle emissions.

In the aerospace industry, where weight reduction is critical for fuel efficiency and performance, narrow reinforcement could be employed in components such as interior panels, cargo compartments, and structural supports. These applications would benefit from the high tensile strength provided by fiber reinforcement, with reduced material requirements helping to keep manufacturing costs manageable. Additionally, the ecological benefits of using natural fibers like jute or hemp align with the aerospace industry’s increasing emphasis on sustainability and environmental responsibility. For the construction industry, the narrow reinforcement approach could be applied to structural elements, such as beams, panels, and connectors, where high tensile strength is required only in specific areas. Using this method in building materials would contribute to sustainable architecture and green building practices by lowering the carbon footprint of materials used in construction. The reduced material use also allows for easier recycling and end-of-life processing, as fewer fiber materials need to be separated and disposed of, supporting a circular economy model.

#### 3.1.5. Advancing Ecodesign with TRIZ/ARIZ and Targeted Reinforcement

This study’s findings align with and support the application of TRIZ (Theory of Inventive Problem Solving) and ARIZ (Algorithm of Inventive Problem Solving) methodologies in advancing ecodesign. The “Local Quality” principle from TRIZ, which promotes the localized application of materials to maximize efficiency, is particularly relevant. By implementing narrow reinforcement, we address the specific need for strength in the high-stress regions of the composite, rather than reinforcing the entire structure unnecessarily. This selective approach not only conserves materials, but also reduces the overall weight of the composite, which is advantageous in applications where weight reduction is essential.

Additionally, the economic and ecological advantages of this approach suggest that the TRIZ and ARIZ principles could be further explored to optimize composite designs for various industries. Future studies could investigate additional fiber configurations, such as modular or segmented reinforcements, where fibers are strategically placed only in critical load-bearing regions. Such designs would allow for even greater resource efficiency and could potentially lead to new composite structures that are easier to manufacture, maintain, and recycle. Incorporating this targeted reinforcement strategy into standard manufacturing practices also has implications for reducing environmental impact on a global scale. As industries increasingly prioritize sustainable materials and processes, the shift to fiber-reinforced composites that require fewer resources and yield lower emissions can support global efforts to combat climate change. The findings of this study lay the groundwork for a new generation of eco-efficient composite materials, demonstrating that high mechanical performance can be achieved without compromising environmental goals.

### 3.2. SEM Analysis for Composite Materials

#### 3.2.1. SEM Analysis for Glass Fiber Composites

In our SEM analysis of glass fiber composites, captured using a Phenom Pure G6 Desktop SEM microscope, we examined the fracture surfaces to assess microstructural characteristics and failure modes. The SEM image at 980× magnification reveals insights into fiber–matrix adhesion, void distribution, and fracture behavior, which are pivotal for understanding the mechanical performance of these composites under tensile stress, as can be seen in Figure 11 below.

1 Fiber–Matrix Adhesion: The SEM image shows varying degrees of adhesion between the glass fibers and the polymer matrix. Some fibers exhibit strong bonding, while others reveal slight gaps, indicating inconsistent adhesion quality. Poor adhesion, indicated by visible gaps, can weaken the composite’s overall tensile strength, as it hinders the effective transfer of stress from the matrix to the reinforcing fibers. Enhanced bonding between the fibers and matrix could potentially improve load distribution and resistance to tensile forces.
2 Presence of Voids and Defects: The image highlights the absence of voids but shows occasional fiber pull-out. Fiber pull-out typically indicates weaker interfacial adhesion in certain areas, which can reduce the composite’s ability to absorb energy during fracture. These defects impact the durability and load-bearing capacity, especially under tensile conditions, emphasizing the importance of optimizing fiber–matrix adhesion to enhance performance.
3 Fiber Orientation and Distribution: The glass fibers exhibit a somewhat random orientation within the matrix. This alignment is advantageous for achieving isotropic mechanical properties, as it allows the composite to resist forces from multiple directions. Effective fiber distribution within the polymer matrix, as shown here, enhances the composite’s versatility in applications that require multi-axial strength.
4 Fracture Surface Morphology: The SEM image displays rough, uneven fracture surfaces on the polymer matrix, with visible lines and breakage points, typical of brittle failure modes. This morphology suggests that the composite matrix is rigid but susceptible to brittle fracture under tensile loads. The combination of fiber breakage and pull-out observed in the image reflects a mixed-mode failure, where both fiber–matrix detachment and fiber fracture contribute to the overall failure mechanism. This combination is often beneficial for toughness, as it allows the composite to absorb more energy during failure.

#### 3.2.2. SEM Analysis for Hemp Fiber Composites

**Figure 12:**
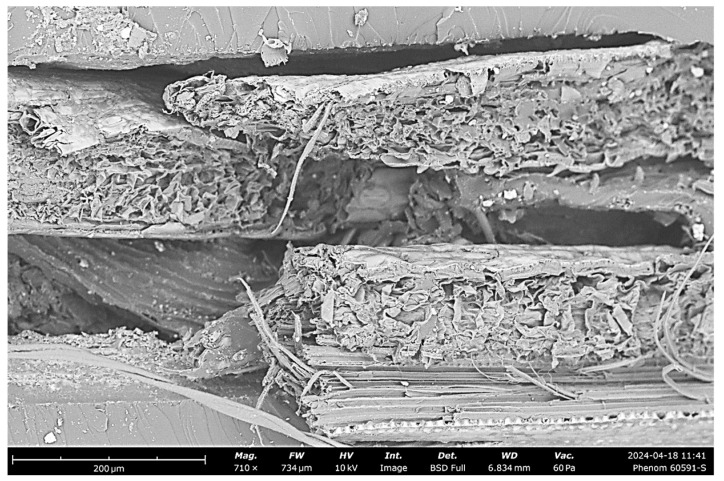
Microstructural analysis of 30% hemp fiber-reinforced polymer composites through SEM.

This analysis focuses on the fiber–matrix interface, fracture morphology, and the microstructural features influencing the composite’s mechanical performance under tensile stress. Figure 12 contains an image captured during SEM analysis.

1 Fiber–Matrix Adhesion: The image reveals distinct fiber–matrix interfaces, with many hemp fibers showing good adhesion to the surrounding polymer matrix. In some regions, however, gaps can be seen between the fibers and matrix, suggesting areas where adhesion may be weaker. Strong fiber–matrix adhesion is essential for effective stress transfer, and inconsistencies here could impact the composite’s overall strength and toughness. The observed gaps may act as stress concentrators and reduce the composite’s ability to withstand tensile loads.
2 Fiber Pull-out and Breakage: A notable characteristic in this image is the presence of extensive fiber pull-out, where fibers are partially detached from the matrix, as well as fractured fiber ends. Fiber pull-out can indicate weaker interfacial bonding, al-lowing fibers to detach instead of breaking. In some regions, broken fiber ends are visible, indicating areas where interfacial bonding was strong enough to cause fiber rupture rather than pull-out.
3 Void Presence and Defects: The SEM image also shows voids within the polymer matrix, visible as dark areas among the fibers. These voids could be a result of air entrapment during fabrication or inconsistencies in resin distribution. The presence of voids is a common manufacturing challenge in natural fiber composites and can significantly impact their mechanical properties.
4 Layered Fiber Structure and Orientation: Hemp fibers appear to be arranged in a somewhat layered structure, with visible orientation across multiple layers. This organization aids in providing strength in specific directions, though it may limit the composite’s isotropic properties compared to more randomly oriented fibers.
5 Fracture Surface Morphology: The fracture surface exhibits rough, irregular textures with visible fiber bundles, typical of brittle failure in natural fiber composites. The layered, fibrous structure observed indicates a mixed-mode failure, where both matrix cracking and fiber pull-out occur, allowing for energy dissipation and enhancing toughness.

#### 3.2.3. SEM Analysis for Jute Fiber Composites

**Figure 13:**
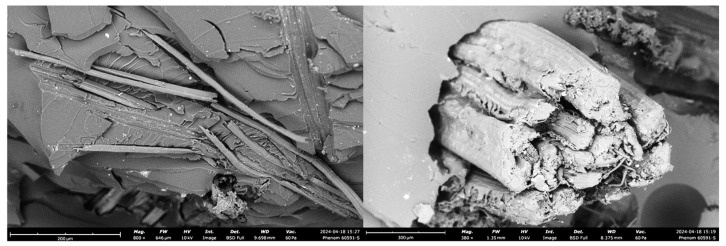
Microstructural analysis of 30% jute fiber-reinforced polymer composites through SEM.

These images from Figure 13 reveal insights into the fiber–matrix adhesion, fracture mechanisms, and the overall structural behavior of the composite under tensile loading, which are critical for understanding jute’s performance in reinforcing polymer matrices.

1 Fiber–Matrix Adhesion: The SEM images highlight the adhesion quality between the jute fibers and the polymer matrix. Some regions display tight bonding with the matrix, while others reveal separation at the fiber–matrix interface. This variability in adhesion can influence load transfer efficiency.
2 Fiber Pull-out and Fracture Behavior: The left image reveals extensive fiber pull-out, where jute fibers are partially detached from the matrix, leaving visible cavities. This pull-out behavior indicates weaker interfacial bonding in specific regions, allowing fibers to slide out under stress instead of breaking. Fiber pull-out can contribute to toughness by absorbing energy during fracture; however, excessive pull-out without sufficient fiber fracture may limit the composite’s tensile strength.
3 Fiber Orientation and Layering: The images reveal a somewhat aligned orientation of jute fibers, especially in the left image, where fibers appear as compact, cylindrical structures rather than as arranged in distinct layers. This alignment enhances strength along the fiber axis, providing directional reinforcement. However, this orientation may reduce isotropic properties, making the composite stronger along the fiber direction but potentially weaker when subjected to multi-directional stress. The compact, cylindrical structure of jute fibers can be advantageous in uniaxial loading applications but may result in inconsistent performance under multi-axial stress due to limited reinforcement in other directions.
4 Void Formation and Structural Defects: The presence of voids is visible in both images, particularly around the fiber–matrix interfaces. These voids are likely formed during the manufacturing process due to trapped air or irregular resin distribution. Minimizing voids during fabrication is crucial for achieving consistent mechanical performance, as they compromise the integrity of the composite and make it more prone to premature failure under tensile loads.
5 Fracture Surface Morphology: The fracture surfaces in the SEM images display rough textures, typical of brittle fracture in the matrix combined with fibrous pull-out. The left image shows fractured surfaces with large, visible gaps, indicating areas where the matrix fails in a brittle manner. The right image shows a fibrous, rough fracture, with fibers protruding from the fractured matrix, demonstrating how fiber pull-out and matrix cracking occur together. This mixed-mode failure, with both fiber detachment and breakage, can increase energy absorption, enhancing toughness but also highlighting the need for improved interfacial bonding to achieve optimal tensile performance.

#### 3.2.4. Summary Table for SEM Analysis

Table 7 presents a concise comparison of the key microstructural observations from the SEM analysis for each fiber-reinforced polymer composite. The analysis focuses on fiber–matrix interface quality, fracture characteristics, void content, and how these factors affect the mechanical performance of the composites. Differences in fiber adhesion and void formation were found to be critical in determining the tensile strength and failure modes of the materials.

### 3.3. Results Obtained forThermogravimetric Analysis (TGA)

Thermogravimetric analysis (TGA) was conducted for all the polymer matrix composite materials: glass fiber, hemp fiber, and jute fiber. Each material was subjected to the same testing parameters as outlined in Table 2, which includes variables such as temperature, heating rate, and holding time.

The resulting TGA graphs display three distinct lines: the TGA line, the DTG line, and the temperature line. The TGA line represents the percentage of mass remaining in the sample as the temperature increases, providing insights into the material’s thermal stability and decomposition behavior. The DTG line, or derivative thermogravimetry, shows the rate of mass change relative to temperature, highlighting the temperatures at which the material experiences the most significant mass loss, and thereby identifying critical thermal events. The temperature line indicates the applied temperature profile during the analysis, offering a contextual reference for interpreting the changes observed in the TGA and DTG lines. This detailed thermal analysis is crucial for assessing the thermal behavior and stability of composite materials, which can influence their application in various industries.

#### 3.3.1. TGA Result for the 30% Glass Fiber Composite

**Figure 14:**
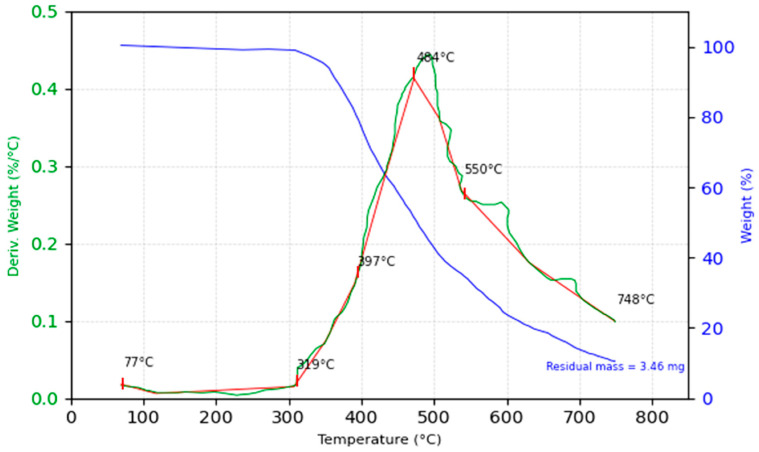
TGA for 30% glass fiber composite.

The thermogravimetric analysis (TGA) of the 30% glass fiber composite reveals its thermal degradation profile and residue formation characteristics under controlled heating. The initial mass of the sample was precisely measured at 43.14 mg, and the analysis was conducted over a temperature range extending to 750 °C, with a focus on identifying key decomposition stages, as shown in Figure 14 below.

Decomposition onset is recorded at 320 °C, marking the initial degradation of the polymer matrix. A rapid mass loss is observed between 350 °C and 590 °C, corresponding to the primary decomposition phase of the epoxy matrix. The temperature at which 50% of the mass is lost occurs at 480 °C, a critical point in understanding the thermal stability of the composite. The maximum decomposition rate, determined from derivative thermogravimetric (DTG) data, coincides with this temperature, emphasizing the degradation of the polymer matrix through chain scission and volatilization processes typical of epoxy systems.

Beyond 480 °C, the mass stabilizes, signifying the near-complete breakdown of the organic matrix. The residual mass is 8.02% (3.46 mg), attributable to the glass fiber fraction, which is known for its high thermal stability. The inorganic nature of the glass fibers ensures no further decomposition occurs up to the maximum temperature of 800 °C, a behavior well documented for glass-reinforced polymer composites. The glass fibers, being thermally inert, provide structural reinforcement even as the matrix degrades.

The TGA results confirm that the glass fiber composite retains its structural integrity at elevated temperatures, with glass fibers contributing significantly to the residual mass. The onset of matrix decomposition at 320 °C and the rapid mass loss phase between 350 °C and 590 °C define the upper thermal limits for practical applications of this composite. Despite the degradation of the polymer matrix, the glass fibers ensure residual stability, an essential factor in high-temperature engineering applications.

#### 3.3.2. TGA Result for the Jute Fiber Composite

**Figure 15:**
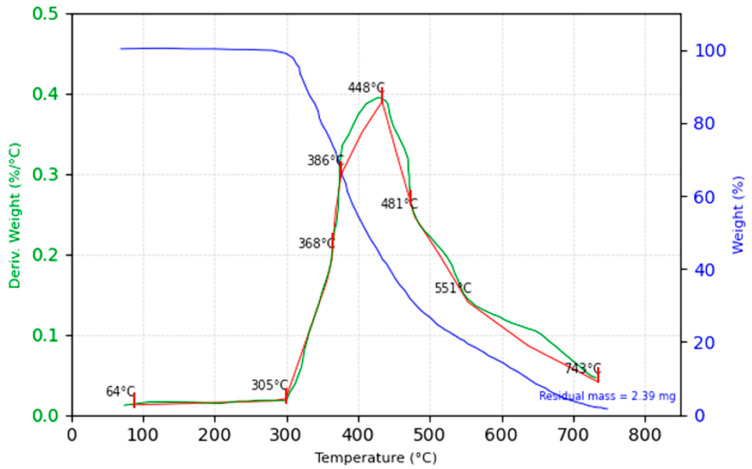
TGA for jute fiber composite.

The TGA of the jute fiber composite, with an initial sample weight of 64.365 mg, reveals several key observations, highlighting the superior qualities of this bio-composite, which can be extracted from Figure 15.

Minimal mass loss is observed up to 300 °C, indicating the composite’s stability within this range and suggesting the absence of significant moisture or volatile content. The onset of decomposition is recorded at 300 °C, marking the initial thermal degradation of the polymer matrix and the organic components of the jute fibers. This decomposition onset is consistent with the degradation of natural fibers and epoxy matrices, transitioning the composite from thermal stability to active degradation.

The most significant thermal event occurs between 350 °C and 550 °C, representing the primary decomposition phase. The temperature at which 50% mass loss occurs is 450 °C, highlighting a critical point in the thermal degradation process. The maximum decomposition rate, as shown by derivative thermogravimetric (DTG) data, occurs at 440 °C, indicating the highest rate of matrix and fiber degradation. This phase involves rapid mass loss, primarily due to the breakdown of cellulose, hemicellulose, and lignin, which are the main constituents of jute fibers.

The thermal decomposition of jute fibers follows a characteristic pattern for lignocellulosic materials. Hemicellulose degrades around 200 °C to 300 °C, followed by cellulose degradation between 300 °C and 350 °C. Lignin, with a broader degradation range, contributes to slower mass loss beyond 350 °C, continuing up to 550 °C. This behavior is typical for natural fibers and results in the major mass loss observed within this temperature range.

After 550 °C, the rate of mass loss slows significantly, with the minimal residual degradation of the polymer matrix and organic residues. By the end of the analysis, the residual mass is 3.71%, equivalent to 2.39 mg, consisting primarily of charred material and ash. This relatively low residual mass underscores the fact that jute fibers, being natural lignocellulosic materials, undergo nearly complete decomposition at moderate temperatures, leaving minimal residue compared to synthetic fiber composites.

The TGA data highlight a two-phase degradation process: an initial stable phase up to 300 °C, followed by the primary degradation phase from 350 °C to 550 °C. This distinct behavior is typical of jute fiber composites, which, while offering moderate thermal stability, degrade significantly at relatively moderate temperatures. The low residual mass further emphasizes the suitability of jute fiber composites for applications where biodegradability and environmental sustainability are prioritized, but where high-temperature durability is not a primary requirement.

These findings confirm the thermal behavior of natural fiber composites, like jute, which are prone to decomposition at moderate temperatures. While thermal resistance is adequate for applications where biodegradability and eco-friendliness are key, synthetic fibers may be more appropriate in high-temperature environments due to their superior thermal stability.

#### 3.3.3. TGA Result for the Hemp Fiber Composite

**Figure 16:**
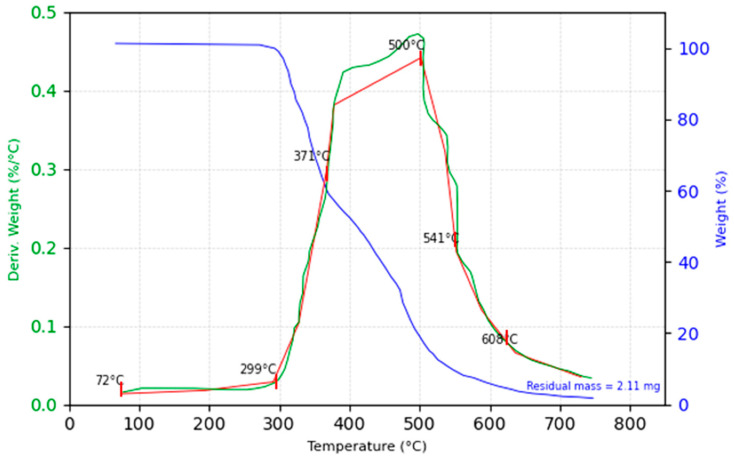
TGA for hemp fiber composite.

The thermogravimetric analysis (TGA) of the hemp fiber composite, starting with an initial sample mass of 57.11 mg, offers a detailed characterization of its thermal stability and decomposition behavior, as seen in Figure 16.

The TGA curve reveals several distinct phases of thermal decomposition. Initially, the composite remains stable up to approximately 305 °C, with minimal mass loss, indicating the absence of significant volatiles or moisture content. The onset of significant decomposition is observed at 305 °C, marking the beginning of the degradation of the organic components within both the hemp fibers and the polymer matrix. This phase of decomposition intensifies between 305 °C and 440 °C, which corresponds to the primary breakdown of cellulose, hemicellulose, and lignin present in the hemp fibers, along with the degradation of the epoxy matrix.

The temperature corresponding to a 50% mass loss is observed at 440 °C, and the maximum rate of decomposition, as indicated by derivative thermogravimetric (DTG) data, occurs at 450 °C. This peak represents the most rapid phase of thermal degradation, primarily driven by the breakdown of the hemp fibers and the polymer matrix.

Beyond 450 °C, the decomposition rate slows significantly as the majority of the organic material has been degraded. The TGA curve stabilizes, indicating that most of the combustible material has been exhausted. By the end of the analysis, at approximately 500 °C, the residual mass is recorded at 3.69%, equivalent to 2.11 mg. This residue consists primarily of char and inorganic materials, likely derived from the incomplete combustion of the hemp fibers and any inorganic fillers or additives present in the composite.

The relatively low residual mass underscores the fact that hemp fibers, while prone to decomposition at moderate temperatures, leave behind a small amount of char. This residue provides some degree of thermal stability, but the overall decomposition behavior reflects the organic nature of the hemp fibers, which undergo nearly complete thermal degradation within the specified temperature range.

The TGA and DTG data confirm a two-phase degradation process for the hemp fiber composite. The first phase exhibits thermal stability up to 305 °C, while the second phase, between 305 °C and 440 °C, is characterized by the rapid decomposition of both the polymer matrix and the fibers. The residual mass after 500 °C is primarily char, reflecting hemp’s limited thermal stability. These findings suggest that hemp fiber composites are suitable for applications requiring moderate thermal resistance, but are less appropriate for high-temperature environments, where greater thermal stability is critical.

#### 3.3.4. Thermogravimetric Analysis (TGA) Summary and Conclusions

Table 8 below summarizes the TGA results for each composite material.

- Key Observation:

1. Thermal Stability:

The glass fiber composite demonstrates the highest thermal stability, with a decomposition onset at 320 °C and a final residual mass of 8.02%. The non-combustible nature of glass fibers makes this composite suitable for high-temperature applications, such as automotive components, where thermal resistance is critical.

Natural fiber composites, hemp and jute, exhibit lower thermal stability, with decomposition onsets at 305 °C and 300 °C, respectively. Although their thermal stability is lower than glass, these natural fibers are better suited for eco-friendly applications where biodegradability is a priority.

2. Decomposition Behavior:

The primary decomposition phase for glass fiber composites occurs from 350 °C to 590 °C, while hemp and jute fibers decompose mainly between 380–510 °C and 350–550 °C, respectively. The natural fibers’ decomposition involves the breakdown of cellulose and lignin, peaking around 450 °C (hemp) and 440 °C (jute), resulting in minimal residual mass post-degradation.

Jute shows slightly better decomposition performance among natural fibers, with a higher decomposition temperature range, suggesting moderate thermal stability suitable for structural applications with eco-friendly requirements.

3. Residual Mass:

The residual mass after decomposition is highest for glass fiber composites (8.02%) due to the inorganic nature of the glass fibers, which do not degrade. This feature supports applications that require structural integrity retention at elevated temperatures.

Hemp and jute leave a low residual mass (3.69% and 3.71%, respectively), which aligns with their suitability for biodegradable applications, though it limits their use where thermal residue retention is essential.

## 4. Discussion

The present study aimed to enhance the mechanical properties of polymeric resins through reinforcement with both synthetic and natural fibers, including glass, hemp, and jute fibers. The key objective was to achieve material performance improvements while maintaining economic and ecological sustainability, using TRIZ-ARIZ principles to optimize reinforcement. The findings reveal significant enhancement in tensile properties through targeted fiber placement, positioning our research within a growing body of literature on fiber-reinforced composites. This discussion presents a clear comparison between our contributions and the work of previous studies, emphasizing the innovations, differences, and broader implications of our approach.

Previous studies have broadly examined the application of natural fibers in polymer composites, focusing on enhancing mechanical properties and environmental benefits. Notably, Refs. [18,26,30] explored natural fiber-reinforced composites (NFCs) and highlighted several advantages, including biodegradability, low cost, and eco-friendliness. However, these composites also have limitations, such as high moisture absorption and low compatibility with polymer matrices [15]. Our study aimed to address these challenges by employing targeted fiber reinforcement and control of air humidity and fiber humidity, a simple approach compared to other chemical methods used in earlier research. By integrating TRIZ-ARIZ principles, we improved both material efficiency and mechanical performance, addressing key issues that have historically limited the applicability of natural fiber composites.

Other studies like [1] or [31] focus on enhancing the fiber–matrix adhesion by employing surface treatments, such as alkali and silane treatment, to reduce the hydrophilic properties of natural fibers. This study concluded that chemical treatment improved the bonding characteristics between the fiber and the polymer, leading to enhanced tensile properties. In our study, considering that this treatment could endanger and inhibit the recyclability as was already proven in other studies [43], similar treatment techniques were not used, but an optimization of the reinforcement placement strategy instead. We observed that targeted reinforcement—where fibers are applied selectively to areas under the greatest stress—resulted in comparable tensile strength improvements while significantly reducing material usage. This approach offers a practical solution for minimizing costs and improving the ecological perspective while maintaining performance.

A critical difference between our study and other studies, such as [24,26], is our focus on targeted reinforcement using TRIZ-ARIZ methodologies. In these studies, the authors used a hybridization of natural fibers with synthetic fibers (such as flax and glass) to achieve improved mechanical properties. Although this hybridization led to improved mechanical and thermal properties, it required more extensive use of reinforcement material and synthetic components. In contrast, we adopted an approach that prioritizes both ecological and economic sustainability by reducing the overall material use without compromising on tensile strength. Our findings suggest that strategic fiber placement can achieve a similar outcome to full-length reinforcement, providing an optimal ecodesign solution for practical applications where reducing environmental impact is big.

In the present study, we demonstrated that natural fibers such as jute and hemp can effectively enhance the tensile properties of polymeric resins, which aligns well with previous research that has emphasized the potential of these fibers. Refs. [13,31,44] highlighted that polymeric matrix composites and even PLA reinforced with jute and nettle fibers achieved enhanced tensile strength and Young’s modulus compared to non-reinforced PLA composites. Similarly, our results indicate that jute and hemp fibers contribute significantly to tensile improvements, particularly when applied in a targeted reinforcement configuration. However, our study diverges from Chaudhary et al.’s work [44] by demonstrating that targeted fiber reinforcement, combined with strategic placement, achieves material savings of up to 20%, thereby contributing to a more economically feasible solution.

The literature also suggests that synthetic fibers like glass and carbon fibers offer superior mechanical properties, such as high tensile and impact strength, compared to natural fibers. The study [16] explored glass–carbon composites and emphasized their high mechanical performance and durability. Our results align with these findings regarding synthetic fibers, but we also highlighted a significant environmental drawback—namely, the non-biodegradable nature of synthetic reinforcements and the associated carbon footprint. By reinforcing polymeric resins with natural fibers instead of synthetic fibers, our study directly addresses environmental concerns and demonstrates a promising pathway to replacing synthetic fibers in many applications, especially where environmental impact reduction is a priority.

Sustainability was a core aspect of the current research, driving the exploration of natural fibers in composite materials. Numerous prior studies have examined the potential of natural fibers to reduce environmental burdens. For instance, Refs. [8,19] highlighted the increasing use of plant-based natural fibers in place of conventional synthetic fibers, noting that they reduce greenhouse gas emissions and are generally more sustainable. Our study extends this focus on sustainability by employing TRIZ-ARIZ principles to optimize reinforcement placement and reduce material usage, thus minimizing both environmental impact and economic costs. This aligns with the eco-design perspective advocated by [3], the authors of which emphasized the importance of optimizing the use of materials throughout the design, manufacturing, and end-of-life stages of product development.

Our approach to ecodesign also incorporates targeted reinforcement to minimize resource consumption while retaining high mechanical performance. Previous research, such as [34], has focused on hybrid composites, which mix natural and synthetic fibers to achieve enhanced properties. These hybrid composites generally offer improved mechanical performance, but at the cost of increased environmental impact due to the inclusion of synthetic fibers. In contrast, our study focuses exclusively on natural fiber reinforcements and aims to close the performance gap through optimized fiber placement rather than hybridization. This approach has implications for industries such as automotives and construction, where reducing material waste and maximizing sustainability are critical considerations.

The importance of ecodesign principles is further corroborated by studies like [15,45,46], which emphasize that natural fibers such as kenaf and sisal could be sustainably integrated into polymer matrices for enhanced mechanical properties. Similarly, Ref. [47] analyzed the environmental impact of using flax fibers and found that such natural fibers contributed significantly to reducing overall emissions and resource usage in composite manufacturing. Our work builds on these findings by not only incorporating natural fibers but also optimizing their usage through targeted reinforcement strategies to further enhance sustainability.

Future research could explore the potential for hybrid composites in cases where higher performance is required, while still maintaining a focus on sustainability by limiting the proportion of synthetic materials. Additionally, our findings suggest that advanced additive manufacturing techniques, such as 3D printing, could provide additional opportunities for optimizing fiber placement, allowing for even greater customization of composite materials for specific mechanical applications. Research into the long-term durability of these materials, especially with respect to environmental factors such as UV exposure and biodegradation, could also provide valuable insights into their practical applications in the field.

Research by the authors of [19] highlighted the role of eco-friendly resins in enhancing the sustainability of natural fiber composites, emphasizing that bio-based resins could further reduce the environmental footprint of such materials. Integrating bio-based matrices with optimized fiber placement, as done in our study, could provide an even more sustainable solution for industries aiming to reduce their reliance on petrochemical-based materials.

In terms of potential applications, Refs. [48,49] demonstrated the viability of natural fiber composites in automotive interiors, where reduced weight and environmental sustainability are key concerns. Similarly, Ref. [11] showed that hemp fiber composites could achieve significant weight reductions without compromising safety standards, suggesting that such materials could effectively replace synthetic composites in automotive applications. Our findings align with these studies and indicate that targeted reinforcement with natural fibers could provide additional material savings and performance benefits.

The implications of this study are significant for industries seeking to implement sustainable materials in their products, particularly in sectors such as automotion, construction, and consumer goods. The adoption of targeted fiber reinforcement strategies provides an effective way to reduce the carbon footprint and material costs associated with composite materials, aligning with the goals of sustainable development as outlined by the United Nations’ Agenda for Sustainable Development [49]. By demonstrating that natural fibers can achieve comparable tensile strength to synthetic fibers with less material, our approach contributes to the advancement of composite design strategies that prioritize both performance and sustainability.

The use of targeted reinforcement, optimized through TRIZ-ARIZ principles, holds significant promise for reducing the use of synthetic fibers and minimizing material waste. Industries such as the automotive sector, which are moving towards lighter and more sustainable materials, can benefit from this approach by integrating natural fiber composites into their product designs. Our findings suggest that replacing non-renewable glass or carbon fibers with natural fibers like jute or hemp could yield comparable mechanical performance with reduced environmental impact, especially when used in non-structural applications or areas subjected to moderate mechanical stress.

In the construction sector, where sustainability is becoming an increasingly important consideration, the use of natural fiber-reinforced composites could provide a greener alternative to traditional building materials. The enhanced thermal and acoustic properties of natural fibers, as highlighted in one article [8], combined with our findings on tensile reinforcement, suggest that these composites could play an important role in green building initiatives. Additionally, targeted reinforcement offers a practical means to reduce the quantity of material used while maintaining structural integrity, which is particularly relevant in the context of large-scale construction projects.

Furthermore, studies like those by the authors of [32] indicate that natural fibers such as bamboo can improve both the mechanical properties and environmental sustainability of concrete composites when used as a reinforcement material. Our research could be expanded to explore the potential for integrating natural fiber reinforcements into other construction materials, thereby extending the benefits of targeted reinforcement to a broader range of building applications.

## 5. Conclusions

The results indicate that natural fibers such as hemp and jute significantly improve tensile strength, though with some compromise in thermal stability compared to glass fiber composites. Among the fibers tested, jute achieved the highest tensile strength, surpassing both glass and hemp when embedded in the polymer matrix. This was particularly evident in specimens reinforced in narrow-gauge areas where the maximum stress is concentrated during tensile testing. The localized reinforcement strategy proved as effective as full-length reinforcement, achieving comparable tensile strengths while reducing material use by up to 60%. This approach demonstrates both economic and ecological advantages. Selective reinforcement, guided by TRIZ-ARIZ principles, optimized fiber placement, minimized material usage, and reduced overall specimen weight without sacrificing mechanical performance. This strategy is especially advantageous for synthetic fibers like glass, which are more expensive and environmentally taxing. Furthermore, TRIZ segmentation principles enabled modular designs that allow for the easy repair and replacement of damaged sections, promoting reusability and reducing waste. Thermal analysis via TGA revealed that glass fiber composites have superior thermal stability, tolerating temperatures up to 480 °C before significant degradation. This makes them ideal for high-temperature applications, such as aerospace and automotive components. In contrast, natural fibers like jute and hemp began degrading at 300–350 °C, consistent with lignocellulosic materials. Despite this, jute composites offer moderate thermal stability and biodegradability, making them suitable for applications prioritizing environmental impact over thermal endurance. SEM analysis highlighted the quality of fiber–matrix adhesion, which is critical for load transfer efficiency. Glass fibers exhibited excellent adhesion, with minimal voids and cohesive fracture patterns. Jute fibers, despite strong tensile properties, showed gaps at the interface, likely due to residual moisture affecting adhesion. Addressing this challenge through surface treatment or vacuum curing could enhance fiber–matrix compatibility and improve performance. The TRIZ-ARIZ methodology facilitated innovative problem-solving in sustainable material design, addressing ecological constraints without compromising performance. By applying TRIZ principles like “Continuity of Useful Action” and “Segmentation”, modular composite designs with consistent load-bearing capacity, recyclability, and ease of assembly were developed. These attributes are essential for advancing green engineering solutions.

In conclusion, this study offers a framework for designing economically and ecologically viable polymer composites with improved mechanical and thermal properties. The use of synthetic and natural fibers, optimized through TRIZ-ARIZ principles, achieved a balance of tensile strength, material efficiency, and environmental responsibility. Future research could explore hybrid composites combining natural and synthetic fibers, as well as finite element analysis to refine reinforcement placement. This work highlights the potential of TRIZ-ARIZ methodologies in driving sustainable innovation in material engineering, addressing the growing need for lightweight, durable, and environmentally friendly materials.

## Figures and Tables

**Table 1:** TGA test parameters.

Nr. Crt.	Temperature [°C]	Heating Rate [°C/min]	Holding Time [min]
1	250	35	1
2	450	20	1
3	750	20	0

**Table 2:** Tensile strength results for full and partial reinforcement [MPa].

Fiber Type	Fiber Positioning	Maximum Tensile Strength	Average Tensile Strength	Standard Deviation	Module
None	N/A	19.21	18.43	0.5048	18
Glass	Narrow Area	34.55	34.73	0.6332	34
	Whole Part	34.52	34.76	0.7348	33
Hemp	Narrow Area	24.98	24.54	0.8945	24
	Whole Part	24.86	24.66	0.6462	23
Jute	Narrow Area	43.45	41.98	0.7082	41
	Whole Part	43.54	42.95	0.5815	42

**Table 3:** Mechanical performance for fully and partially reinforced composites.

Fiber Type	Fiber Positioning	Young’s Modulus (GPa)	Strain-to-Failure (%)
None	N/A	2.1	1.5
Glass	Narrow Area	5.8	2.3
	Whole Part	5.7	2.4
Hemp	Narrow Area	3.9	1.9
	Whole Part	3.8	2.0
Jute	Narrow Area	7.2	2.8
	Whole Part	7.1	2.9

**Table 4:** Tensile strength results for three-part assembly test specimen [MPa].

Fiber Type	Fiber Positioning	Maximum Tensile Strength	Average Tensile Strength	Standard Deviation	Module
Glass	Puzzle	22.97	22.23	0.5488	21
	Dovetail	20.68	20.27	0.3434	20
	T-channel 1	20.77	20.37	0.4935	20
	T-channel 2	21.26	20.85	0.3898	21
Hemp	Puzzle	19.51	18.97	0.4685	19
	Dovetail	17.11	16.43	0.7774	17
	T-channel 1	16.11	15.21	0.4980	15
	T-channel 2	16.60	16.41	0.1318	16
Jute	Puzzle	22.80	22.42	0.3525	22
	Dovetail	20.20	19.23	0.6878	19
	T-channel 1	20.25	19.82	0.3017	20
	T-channel 2	20.34	19.76	0.5397	19

**Table 5:** Tensile strength results for the ribs improved three-part assembly test specimen [MPa].

Fiber Type	Fiber Positioning	Maximum Tensile Strength	Average Tensile Strength	Standard Deviation	Module
Glass	Puzzle with ribs	26.87	26.05	0.63	25
	Dovetail with ribs	24.18	23.49	0.43	23
	T-channel 1 with ribs	24.30	23.82	0.44	24
	T-channel 2 with ribs	25.30	24.52	0.67	23
Hemp	Puzzle with ribs	23.01	22.23	0.64	21
	Dovetail with ribs	20.01	19.21	0.71	18
	T-channel 1 with ribs	18.52	17.75	0.45	17
	T-channel 2 with ribs	19.21	19.02	0.17	19
Jute	Puzzle with ribs	26.90	26.24	0.57	25
	Dovetail with ribs	23.63	22.47	0.98	21
	T-channel 1 with ribs	23.89	23.38	0.43	23
	T-channel 2 with ribs	23.61	22.95	0.47	22

**Table 6:** Tensile test results for anti-action specimens [MPa].

Fiber Type	Fiber Positioning	Maximum Tensile Strength	Average Tensile Strength	Standard Deviation	Module
Glass	Puzzle AA	15.24	14.62	0.39	14
	Dovetail AA	13.23	12.89	0.25	13
	T-channel 1 AA	13.49	13.15	0.36	13
	T-channel 2 AA	13.87	13.42	0.42	13
Hemp	Puzzle AA	13.26	12.41	0.72	12
	Dovetail AA	11.29	10.67	0.51	10
	T-channel 1 AA	10.35	9.96	0.30	9
	T-channel 2 AA	10.78	10.43	0.32	10
Jute	Puzzle AA	15.07	14.39	0.53	14
	Dovetail AA	13.30	12.65	0.64	12
	T-channel 1 AA	13.01	12.68	0.26	12
	T-channel 2 AA	13.43	13.07	0.33	13

**Table 7:** Overview table with SEM key findings.

Fiber Type	Fiber–Matrix Interface	Fracture Mode	Void Content	Fiber Characteristics	Overall Mechanical Impact
Glass Fiber	Mixed adhesion, with visible gaps and some areas of pull-out	Brittle fracture with fiber breakage in well-bonded areas, pull-out in weakly bonded areas	No visible void	Random orientation, consistent diameters	Moderate tensile strength; inconsistent bonding reduces load transfer efficiency
Hemp Fiber	Insufficient adhesion, smooth fiber surfaces indicating slippage	Irregular fracture surfaces, internal fiber bundle separation	Moderate, voids due to incomplete resin infiltration	Layered structure, non-uniform distribution	Limited tensile strength due to variable bonding and voids, though fiber pull-out contributes to toughness
Jute Fiber	Variable adhesion, with fiber debonding and partial fiber pull-out	Brittle failure, fiber pull-out, minimal plastic deformation	High, non-porous structure leads to complete resin impregnation	Compact cylindrical fibers, non-layered, well-packed	Low mechanical performance, poor fiber–matrix interaction, and voids

**Table 8:** TGA investigation key findings for composite materials.

Composite Material	Initial Mass (mg)	Final Mass (mg)	Residual Mass (%)	Starting Decomposition Temp (°C)	Temperature at 50% Mass (°C)	Main Decomposition Temp Range (°C)
Glass Fiber	43.14	3.46	8.02%	320 °C	480 °C	350–590 °C
Hemp Fiber	57.11	2.11	3.69%	305 °C	440 °C	380–510 °C
Jute Fiber	64.37	2.39	3.71%	300 °C	450 °C	350–550 °C

## Data Availability

The original contributions presented in this study are included in the article material. Further inquiries can be directed to the corresponding authors.
